# Kazak faecal microbiota transplantation induces short-chain fatty acids that promote glucagon-like peptide-1 secretion by regulating gut microbiota in *db/db* mice

**DOI:** 10.1080/13880209.2021.1954667

**Published:** 2021-08-15

**Authors:** Xue Han, Ye Wang, Peipei Zhang, Manli Zhu, Ling Li, Xinmin Mao, Xiaoting Sha, Linlin Li

**Affiliations:** a Department of Pharmacology, Xinjiang Medical University, Urumqi, China; b College of Pharmacy, Heze University, Heze, China; c Central Laboratory of Xinjiang Medical University, Xinjiang Medical University, Urumqi, China; d Chemical Department, Pharmacy College, Xinjiang Medical University, Urumqi, China; e State Key Laboratory of Pathogenesis, Prevention and Treatment of High Incidence Diseases in Central Asia of Xinjiang Medical University, Urumqi, China; f Department of Traditional Chinese Medicine, Xinjiang Medical University, Urumqi, China; g Key Laboratory of Active Components of Xinjiang Natural Medicine and Drug Release Technology, Urumqi, China

**Keywords:** Kazak individuals, type 2 diabetes mellitus, short-chain fatty acids-producing bacteria

## Abstract

**Context:**

Faecal microbiota transplantation (FMT) from Kazak individuals with normal glucose tolerance (KNGT) significantly reduces plasma glycolipid levels in type 2 diabetes mellitus *db/db* mice. However, the mechanism behind this effect has not been reported.

**Objective:**

To study the mechanism of improved glycolipid disorders in *db/db* mice by FMT from a KNGT donor.

**Materials and methods:**

The normal diet group consisted of *db/m* mice orally administered 0.2 mL phosphate buffer saline (PBS) (*db/m + PBS*). For the *db/db + PBS* (Vehicle) and *db/db + KNGT* (FMT intervention group) groups, *db/db* mice received oral 0.2 mL PBS or faecal microorganisms from a KNGT donor, respectively. All mice were treated daily for 0, 6 or 10 weeks. Faecal DNA samples were sequenced and quantified using 16S rRNA gene sequencing and RT-qPCR, respectively. Short-chain fatty acid (SCFA) levels in the mouse faeces were determined by gas chromatography. G protein-coupled receptor 43 (GPR43) and glucagon-like peptide-1 (GLP-1) expression levels were determined.

**Results:**

FMT intervention significantly increased the relative abundance of *Bacteroides uniformis* (0.038%, *p* < 0.05). *Clostridium* levels (LogSQ) were increased (*p* < 0.01), while *Mucispirillum schaedleri* levels (LogSQ) were decreased (*p* < 0.01). Acetate and butyrate levels in the faeces were significantly increased (acetate; butyrate: 22.68 ± 1.82 mmol/L; 4.13 ± 1.09 mmol/L, *p* < 0.05). GPR43 mRNA expression and GLP-1 protein expression increased in colon tissue (*p* < 0.05).

**Discussion and conclusions:**

Mechanistically, FMT-KNGT could improve glycolipid disorders by changing the bacterial composition responsible for producing SCFAs and activating the GPR43/GLP-1 pathway.

## Introduction

The increased prevalence of type 2 diabetes mellitus (T2DM) and its sequelae pose a serious challenge to global health. We previously conducted an epidemiological survey in the Xinjiang Uygur district and showed that the prevalence of T2DM amongst Kazak individuals was 1.56%, significantly lower than that of Uygurs (8.42%) (Tao et al. 2008; Wang et al. 2010). However, the reason for this low prevalence in Kazaks remains unclear. Identifying this reason could be of great significance for T2DM treatment. Recent studies have suggested that T2DM may be due to an imbalance in gut microbial composition and structure (Ma et al. 2018). For example, *Prevotella copri* and *Bacteroides vulgatus* contribute to insulin resistance, while *Akkermansia municiphila* and *Faecalibacterium prausnitzii* increase insulin sensitivity (Li et al. 2016).

Our study showed significant gut differences between individuals with normal glucose tolerance (NGT) and T2DM (Wang et al. 2017). Disturbances in gut microbiota can lead to changes in the type and proportion of short-chain fatty acids (SCFAs) generated in the gut, which are closely related to obesity and T2DM (Qin et al. 2012). SCFAs, especially acetate, propionate, and butyrate, are the main products of intestinal microbial fermentation (Cummings et al. 1987). Interestingly, a previous study revealed that SCFAs improved glycolipid disorders in rodents (Koh et al. 2016). SCFAs act as signal transduction molecules that bind to and activate G protein-coupled receptors (GPRs), such as GPR43 and GPR41 (also called free fatty acid receptors 2 and 3, respectively [FFAR2 and FFAR3]) (Lu et al. 2016). A study focussing on SCFA target molecules in *ffar2*^–/–^ and *ffar*3^–/–^ mice showed that GPR43 and GPR41 were mainly activated by SCFAs, which promoted GLP-1 and peptide-yy (PYY) secretion from L cells and improved glucose tolerance (Samuel et al. 2008; Tolhurst et al. 2012).

In a parallel study, faecal microbiota transplantation (FMT) material from an obese adult twin was performed on germ-free mice, resulting in mouse weight gains and increased obesity-associated metabolic phenotypes compared to FMT using faecal material from the thin twin (Ridaura et al. 2013). We previously treated *db/db* mice with FMT from Kazak individuals with normal glucose tolerance (FMT-KNGT). Our data showed that this FMT improved glucose and lipid metabolism in *db/db* mice, decreased *Desulfovibrio* and *Clostridium coccoides* levels, and increased *Akkermansia municiphila* levels (Zhang et al. 2020). To further explore this hypothesis from a mechanistic perspective, we investigated FMT-KNGT-mediated molecular mechanisms underlying these glucose and lipid metabolic improvements in *db/db* mice.

## Materials and methods

### Ethics statement

The use of human subjects and the *db/db* mouse model was approved by the Ethics Committee of the First Affiliated Hospital of Xinjiang Medical University (Urumqi, China; approval No: 20140212-113) (Supplementary 1 and 2). All participants provided written consent prior to the study (Supplementary 3).

### Preparation of donor faecal fluid and plasma glycolipid analysis

The criteria for KNGT donor selection, preparation of donor faecal fluid, and plasma glycolipid analysis were previously described (Zhang et al. 2020). Briefly, fresh stool from a KNGT individual was collected, sealed quickly and transferred on ice to an aseptic biosafety cabinet. After the addition of sterile saline, the suspension was stirred, passed through stainless steel filters and centrifuged. The sediment was diluted with 0.1 M phosphate buffer saline (PBS, pH 7.2) containing sterile 10% medical-grade glycerine and stored at −80 °C. Preparations were thawed at 37 °C. The study consisted of three groups: *db/m + PBS* (*n* = 9 *db/m* mice), *db/db + PBS* (*n* = 9 *db/db* mice) and *db/db + KNGT* (*n* = 9 *db/db* mice). Faecal suspensions or PBS (0.2 mL) were administered by gavage to *db/db* mice every day for 0, 6 or 10 weeks. The normal control *db/m* mice received PBS. Three mice from each group were euthanized after 0, 6 or 10 weeks of FMT. Fasting blood glucose (FBG) and postprandial glucose (PPG) were measured using the glucose oxidase method. Total cholesterol (TC) and triglyceride (TG) levels were analyzed using the COD-PAP and GPOPAP assays. High-density lipoprotein-cholesterol (HDL-C) and low-density lipoprotein-cholesterol (LDL-C) plasma levels were determined using enzyme-linked immunosorbent assay (ELISA) kits.

### Faecal stool collection and DNA extraction

Faecal samples were collected from all mice after 0, 6 and 10 weeks of FMT. The samples collected at six weeks were also used for 16S rRNA gene sequencing (*n* = 6 per group). The samples were stored at −80 °C until required. DNA was extracted from 200 mg of stool using the QIAamp DNA Stool Mini Kit (Qiagen, Valencia, CA, USA), according to the manufacturer’s instructions. DNA concentration and quality were determined using a NanoDrop 2000 spectrophotometer (Thermo Scientific, Rockford, IL, USA).

### Amplification and sequencing of the V4 region of 16S rRNA gene

All qualified faecal DNA samples were used to construct libraries. The V4 region of the 16S rRNA gene was amplified by polymerase chain reaction (PCR). The libraries were sequenced in paired-end mode on a MiSeq System with sequencing strategy PE 250. The databases used for species annotation based on the 16S rRNA gene for bacterial and archaea community were Greengenes (default) V201305 (DeSantis et al. 2006) and RDP Release 9 201203 (Cole et al. 2014).

### RT-qPCR of intestinal target bacteria

Faecal DNA was amplified using specific primers (Table 1) using a QuantStudio 6 Flex thermocycler (ABI, USA). PCR products were extracted from 1.5% agarose gels, purified using the AxyPrep DNA Gel Extraction Kit (Biosharp, China), and used as templates for RT-qPCR. RT-qPCR was conducted using the TB Green^TM^ Premix EX Taq^TM^ II kit (TaKaRa, Dalian, China). Reactions were performed in triplicate in a 20 μL reaction volume containing 0.8 µL of each primer, 10 µL Taq DNA polymerase, 6 µL nuclease-free water, 100 ng template DNA (2 μL) and 0.4 µL ROX, according to the TB Green^TM^ Premix EX Taq^TM^ II kit instructions. Faecal DNA was amplified using specific target bacteria primers. The primers for the target genes and purified PCR products were designed and tested by Sangon Biotech (Shanghai). The Basic Local Alignment Search Tool (BLAST) was used to verify their accuracy (https://blast.ncbi.nlm.nih.gov/Blast.cgi) (Supplementary 4).

**Table 1.** RT-qPCR primers for intestinal target bacteria.

Target bacteria	Target gene	Amplicon length (bp)	Primer sequences (5′-3′)	Tm (°C)
			(F, forward; R, reverse)	
*Clostridium* (Da Silva et al. 2018)	*16S rRNA*	86	F: CATCCTGATGACGGTTTCTTAACC	58
			R: GTTGCGGGACTTAACCCA	
*Bacteroides* (Jiang et al. 2014)	*16S rRNA*	202	F: CTGAACCAGCCAAGTAGCG	58
			R: CCGCAAACTTTCACAACTGACTTA	
*Sutterella* (Williams et al. 2012)	*16S rRNA*	234	F: CGCGAAAAACCTTACCTAGCC	62
			R: GACGTGTGAGGCCCTAGCC	
*Bacteroides uniformis* (Tong et al. 2011)	*16S rRNA*	112	F: TCTTCCGCATGGTAGAACTATTA	60
			R: ACCGTGTCTCAGTTCCAATGTG	
*Mucispirillum schaedleri*	*16S rRNA*	178	F: CGAGCGTTGTTCGGAGTGACTG	65
			R: CCAGCCAGATTGCCGCCTTC	
*Faecalibacterium prausnitzii* (Bartosch et al. 2004)	*16S rRNA*	177	F: GATGGCCTCGCGTCCGATTAG	60
			R: CCGAAGACCTTCTTCCTCC	
*Ruminococcus gnavus* (Cao et al. 2016)	*16S rRNA*	103	F: GGACTGCATCGTCCAGAAAG	58
			R: AACGTCAGTCATCGTCCAGAAAG	

### Faecal SCFA detection by gas chromatography (GC)

Faecal acetate, propionate and butyrate levels were measured by GC, as previously described (Wu et al. 2015). Approximately 100 mg of each dry stool sample was weighed, suspended in 0.4 mL methanol (Thermo, USA) and homogenized for about 2 min. The pH was adjusted to 1 to 2 by adding 5 M HCl. The samples were incubated at room temperature for 10 min with occasional shaking. The suspension was transferred to a polypropylene tube and centrifuged for 15 min at 12,000 rpm. Centrifugation was repeated until the supernatant was clear. The supernatants were spiked with the internal standard, 2-ethylbutyric acid (Dr. Ehrenstorfer GmbH, Germany), at a final concentration of 0.2 mM. The samples (1 μL) were then injected into a SHIMADZU GC2010 plus system (Japan) equipped with a flame ionization detector (FID). A high-resolution GC column (IntertCap pure-wax, 30 m × 0.25 mm internal diameter, SHIMADZU) coated with a 0.25 μm film thickness was used. Nitrogen was used as the carrier gas. The initial oven temperature was maintained at 100 °C for 0.5 min. The temperature was raised at 3 °C/min to 130 °C and held at this temperature for 15 min. The temperatures of the FID and injection port were 230 °C. The flow rates for hydrogen, air and nitrogen as carrier gases were 30, 300, and 40 mL/min, respectively. The run time for each analysis was 25 min. Acetate, propionate and butyrate levels in the samples were calculated using an internal standard curve method.

### RNA extraction and GPR43 and GLP-1 RT-qPCR

Total RNA was extracted from mouse colon tissue using TRIzol Reagent (Invitrogen, Carlsbad, CA, USA). cDNA was reverse transcribed using the PrimeScript^TM^ RT reagent Kit (TaKaRa, Japan), according to the manufacturer’s instructions. RT-qPCR was performed using the ABI QuantStudio 6 Flex (Applied Biosystems, Foster City, CA, USA). Target primers were designed by Sangon Biotech Co., Ltd (Shanghai) and tested for efficiency using the Primer-BLAST program (http://www.ncbi.nlm.nih.gov/tools/primer-blast/) (Table 2). Co-amplification with β-actin and invariant internal controls was performed for all samples. Assays were performed in triplicate, and data were normalized to internal standard mRNA levels using the 2^−ΔΔCT^ method.

**Table 2.** RT-qPCR primers for gene expression.

Primer name	Primer sequences (5′-3′)	Resource
	(F, forward; R, reverse)	
GPR43	F: ATCCTCCTGCTTAATCTGACCC	NM_146187
	R: CGCACACGATCTTTGGTAGGT	
GLP-1	F: CAAACCAAGATCACTGACAAGAAAT	Primer bank
	R: GGGTTACACAATGCTAGAGGGA	
β-actin	F: CAACTTGATGTATGAAGGCTTTGGT	Primer bank
	R: ACTTTTATTGGTCTCAAGTCAGTGTACAG	

### Western blot analysis

Total protein was extracted from mouse colon tissue using RIPA lysis buffer containing 1 mM phenylmethylsulfonyl fluoride (PMSF, Thermo, USA). Protein concentrations were determined using the Pierce^TM^ bicinchoninic acid (BCA) Protein Assay kit (Thermo, USA). Protein lysates were subjected to 12% SDS-PAGE and transferred onto polyvinylidene fluoride (PVDF) membranes (Roche, USA). The membranes were blocked with 5% non-fat milk for 2 h at room temperature and washed three times with washing buffer (1 × Tris-buffered saline plus Tween 20 [TBST]). Membranes were incubated with anti-GLP-1 primary antibody (1:1000; Affinity, China) at 4 °C overnight, followed by incubation with secondary anti-rabbit horseradish peroxidase (HRP)-conjugated IgG antibody (Bioss, China) at room temperature for 1 h. Protein bands were visualized using an enhanced chemiluminescence kit (ECL, Biosharp, China). Protein signal intensities were quantified using Image J software. Data were normalized to β-actin. Each experiment was repeated in triplicate.

### Statistical analysis

Statistical analysis was performed using SPSS 19.0 software. Differences between the three groups *(db/m + PBS, db/db + PBS* and *db/db + KNGT*) were evaluated using one-way ANOVA analysis for normally distributed variables and the Kruskal–Wallis test for non-normally distributed variables (e.g., the 16S rRNA gene sequencing). All values were expressed as the mean ± standard deviation (SD). *p* Values <0.05 were considered statistically significant.

## Results

### FMT-KNGT improved blood glucose levels in db/db mice

**Figure 1.**
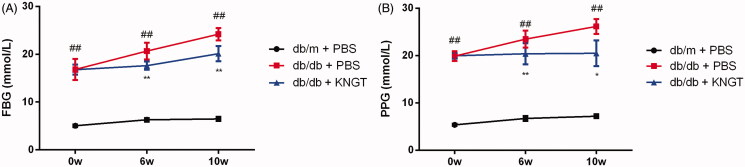
The effects of faecal microbial transplantation from a Kazak individual with normal glucose tolerance on FBG (A) and PPG (B) levels in T2DM db/db mice. Data were analyzed using one-way ANOVA. ## *p* < 0.01 compared to db/m + PBS mice; *p < 0.05; **p < 0.01 compared to db/db + PBS mice.

Comprehensive data from this study are shown in Figure 1 and Table 3. FBG and PPG levels were significantly increased in the mice in the *db/db* + *PBS* group over time (Figure 1(A,B); *p* < 0.01 for both). At 6 and 10 weeks post-FMT, the increases in the FBG and PPG levels were significantly inhibited in the *db/db + KNGT* group (*p* < 0.01 and *p* < 0.05, respectively).

**Table 3.** Effect of FMT-KNGT on blood glucose levels in mice ($\bar{x}$ ± s, mmol/L).

Blood glucose	Group	Time (Week)		
		0w	6w	10w
FBG	db/m + PBS	5.04 ± 0.28	6.28 ± 0.37	6.48 ± 0.42
	db/db + PBS	16.81 ± 2.23##	20.66 ± 1.74##	24.19 ± 1.30##
	db/db + KNGT	16.78 ± 1.05	17.62 ± 0.87**	20.11 ± 1.59**
PPG	db/m + PBS	5.36 ± 0.23	6.72 ± 0.53	7.19 ± 0.40
	db/db + PBS	19.88 ± 1.04##	23.48 ± 1.77##	26.16 ± 1.58##
	db/db + KNGT	19.97 ± 0.65	20.38 ± 2.23**	20.49 ± 2.71*

Data were analyzed using one-way ANOVA. ##*p* < 0.01 compared to *db/m + PBS* mice; *p < 0.05; **p < 0.01 compared to *db/db + PBS* mice. 0w: *n* = 6; 6w: *n* = 6; 10w: *n* = 3.

### The effects of FMT-KNGT on blood lipid levels in db/db mice

**Figure 2.**
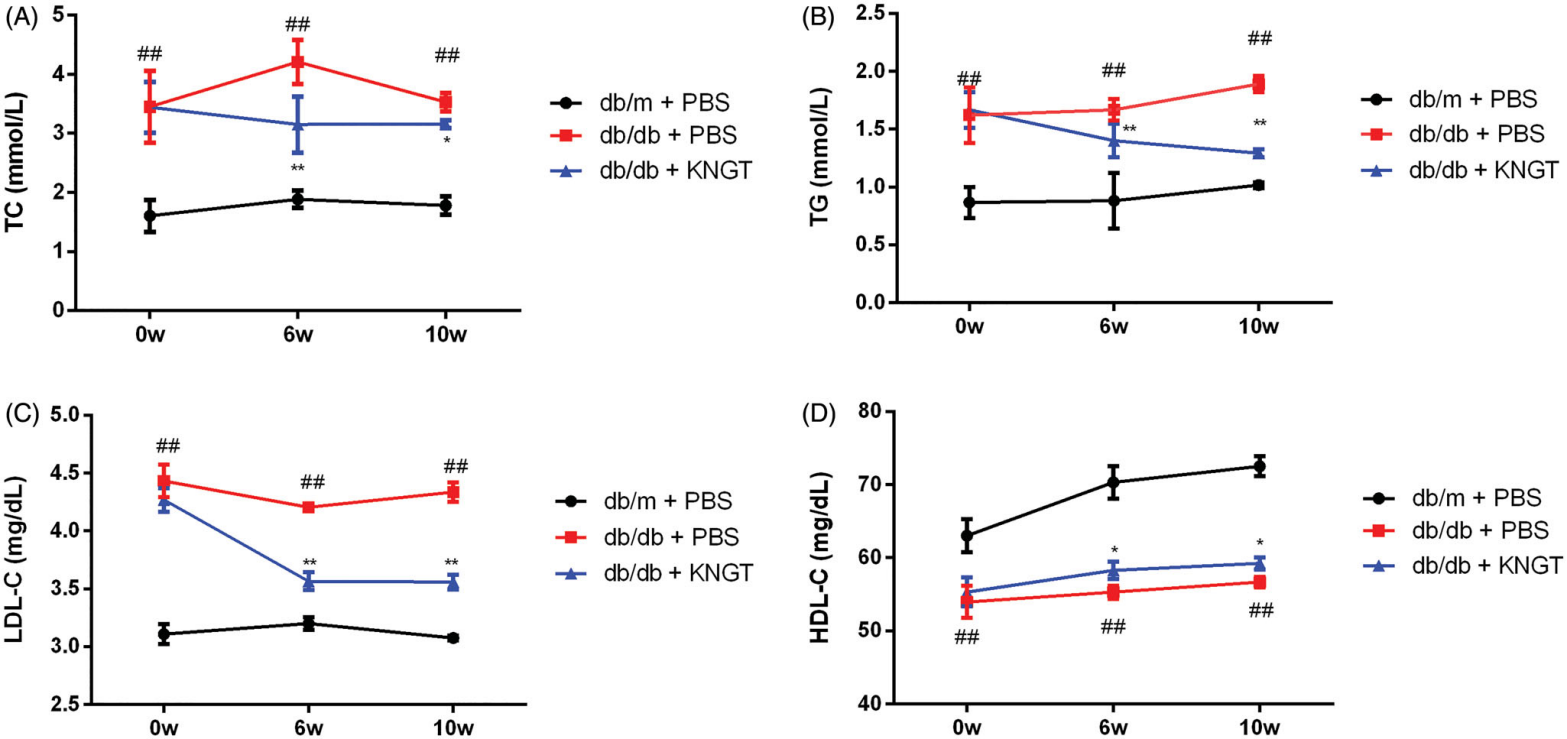
The effects of faecal microbial transplantation from a Kazak individual with normal glucose tolerance on total cholesterol (TC) (A), triglycerides (TG) (B), LDL-C (C) and HDL-C (D) plasma levels in T2DM db/db mice. Data were analyzed using one-way ANOVA. ##*p* < 0.01 compared to db/m + PBS mice; *p < 0.05 and **p < 0.01 compared to db/db + PBS mice.

FMT**-**KNGT improved dysfunctional lipid metabolism in *db/db* mice. As shown in Figure 2 and Table 4, the overall trend of the TC, TG and LDL-C levels in the *db/db + KNGT* group during the post-FMT period demonstrated a slow decline. After six weeks of intervention, the TC, TG and LDL-C levels were significantly reduced compared to the *db/db + PBS* group (*p* < 0.01). In contrast, the HDL-C levels increased significantly (*p* < 0.05) 6 and 10 weeks post-FMT compared to the *db/db + PBS* group (Figure 2(D)).

**Table 4.** Effect of FMT-KNGT on blood lipid levels in mice ($\bar{x}$ ± s, TC, TG: mmol/L; HDL-C, LDL-C: mg/dL).

Blood lipid	Group	Time (week)		
		0w	6w	10w
TC	db/m + PBS	1.61 ± 0.27	1.89 ± 0.15	1.78 ± 0.16
	db/db + PBS	3.45 ± 0.61##	4.21 ± 0.38##	3.53 ± 0.16##
	db/db + KNGT	3.44 ± 0.43	3.15 ± 0.48**	3.16 ± 0.07*
TG	db/m + PBS	0.87 ± 0.13	0.88 ± 0.24	1.02 ± 0.02
	db/db + PBS	1.62 ± 0.24##	1.67 ± 0.09##	1.89 ± 0.07##
	db/db + KNGT	1.67 ± 0.16	1.40 ± 0.14**	1.29 ± 0.04**
LDL-C	db/m + PBS	3.11 ± 0.08	3.20 ± 0.05	3.08 ± 0.02
	db/db + PBS	4.43 ± 0.14##	4.21 ± 0.02##	4.34 ± 0.08##
	db/db + KNGT	4.27 ± 0.10	3.57 ± 0.08**	3.56 ± 0.07**
HDL-C	db/m + PBS	63.02 ± 2.28	70.30 ± 2.21	72.52 ± 1.37
	db/db + PBS	53.97 ± 2.18##	55.30 ± 0.89##	56.68 ± 0.69##
	db/db + KNGT	55.32 ± 1.99	58.28 ± 1.19*	59.24 ± 0.84*

Data were analyzed using one-way ANOVA. ##*p* < 0.01 compared *to db/m + PBS* mice; *p < 0.05; **p < 0.01 compared to *db/db + PBS* mice. 0w: *n* = 6; 6w: *n* = 6; 10w: *n* = 3.

### Intestinal microbiome composition

**Figure 3.**
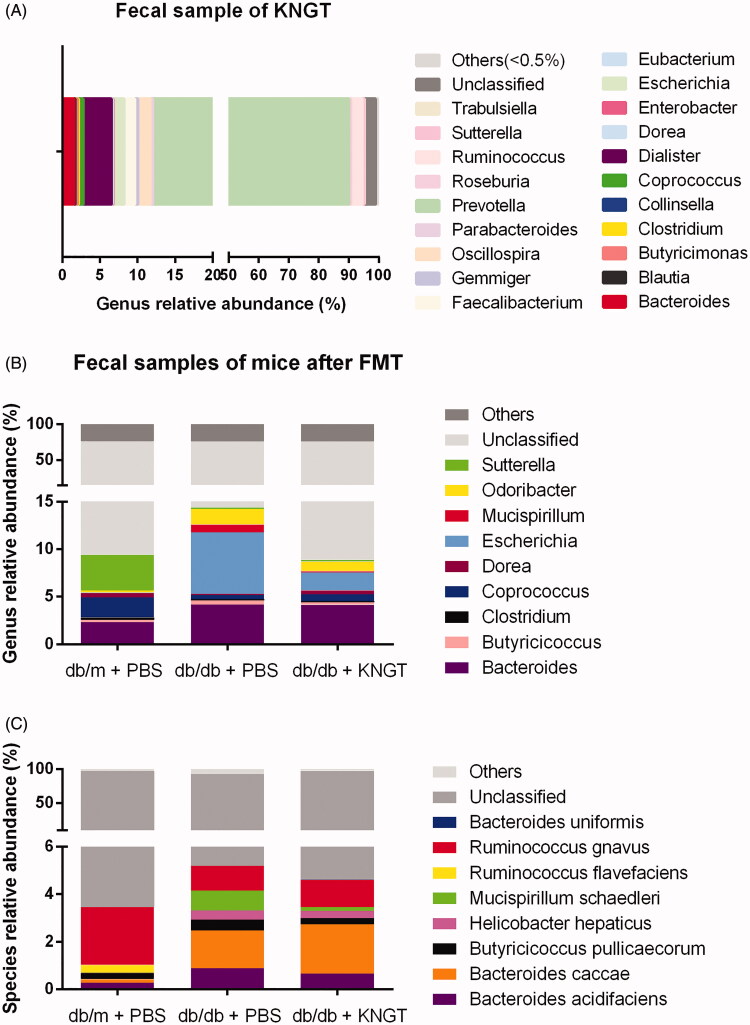
Relative abundance of gut microbiota species at the genus (A) level in faecal samples from the KNGT donor. Relative abundance differences of species at the genus (B) and species (C) levels from each mouse group after FMT-KNGT. Data are presented as percentages. Data were analyzed using the Kruskal–Wallis test. For B and C, mouse faeces were tested at week 6. ‘Others’ refer to bacteria not indicated in the figure.

We performed 16S rRNA gene sequencing on the faeces from the KNGT donor (Figure 3(A), Table 5) and each mouse group after six weeks of FMT (Figure 3(B,C)). The results of the sequencing at the genera and species levels are shown in Tables 6 and 7, respectively. In the KNGT donor stool samples, the relative abundance of the genus *Prevotella* microbes was the highest (78.04%), followed by *Ruminococcus* (3.81%), *Dialister* (3.77%), *Oscillospira* (1.63%), *Bacteroides* (1.53%), and *Escherichia* (1.39%). These bacteria accounted for >90% at the genus level. The remaining bacteria were unclassified or other genera.

**Table 5.** The relative abundance of bacteria in faecal samples from the KNGT donor at the genus level (%).

Bacteria	Relative abundance
*Bacteroides*	1.53
*Blautia*	0.19
*Butyricimonas*	0.28
*Clostridium*	0.13
*Collinsella*	0.01
*Coprococcus*	0.64
*Dialister*	3.77
*Dorea*	0.11
*Enterobacter*	0.11
*Escherichia*	1.39
*Eubacterium*	0.05
*Faecalibacterium*	1.44
*Gemmiger*	0.41
*Oscillospira*	1.63
*Parabacteroides*	0.33
*Prevotella*	78.04
*Roseburia*	0.61
*Ruminococcus*	3.81
*Sutterella*	0.67
*Trabulsiella*	0.05
Unclassified	3.78
Others	1.04

**Table 6.** The relative abundance of the dominant bacteria in faecal samples from mice at the genus level (%, *n* = 6).

Bacteria	db/m + PBS		db/db + PBS		db/db + KNGT	
	Mean	SD	Mean	SD	Mean	SD
*Bacteroides*	2.32	2.2	4.16	2.00	4.14	2.32
*Butyricicoccus*	0.26	0.08	0.45	0.19	0.27	0.3
*Clostridium*	0.25	0.28	0.10	0.14	0.12	0.14
*Coprococcus*	2.10	2.65	0.45	0.36	0.73	0.67
*Dorea*	0.46	0.97	0.14	0.1	0.42	0.71
*Escherichia*	0.03	0.02	6.45	9.68	1.85	0.48
*Mucispirillum*	0.02	0.03	0.83	0.89	0.16	0.25
*Odoribacter*	0.21	0.25	1.66	2.3	1.00	0.96
*Sutterella*	3.78	1.39	0.17	0.18	0.18	0.1
Unclassified	66.13		61.73		66.97	
Others	24.45		23.85		24.14	

Results were obtained using the Kruskal–Wallis test. Mouse faeces were tested at week 6. ‘Others’ refer to bacteria not indicated in the table.

**Table 7.** The relative abundance of the dominant bacteria in faecal samples from mice at the species level (%, *n* = 6).

Bacteria	db/m + PBS		db/db + PBS		db/db + KNGT	
	Mean	SD	Mean	SD	Mean	SD
*Bacteroides uniformis*	0.007	0.004	0.001#	0.001	0.038*	0.04
*Bacteroides acidifaciens*	0.265	0.32	0.885	0.80	0.660	0.43
*Bacteroides caccae*	0.153	0.11	1.591	1.24	2.067	1.91
*Butyricicoccus pullicaecorum*	0.256	0.08	0.453	0.19	0.269	0.3
*Helicobacter hepaticus*	0.017	0.01	0.391	0.29	0.298	0.27
*Mucispirillum schaedleri*	0.021	0.03	0.830	1.89	0.161	0.25
*Ruminococcus flavefaciens*	0.306	0.32	0.000	0.00	0.000	0
*Ruminococcus gnavus*	2.435	3.71	1.044	0.38	1.151	1.27
Unclassified	93.939		87.695		93.064	
Others	2.602		7.110		2.293	

Results were obtained using the Kruskal–Wallis test. Mouse faeces were tested at week 6. ‘Others’ refer to bacteria not indicated in the table. #*p* < 0.05 compared *to db/m + PBS* mice. *p < 0.05 compared to *db/db + PBS* mice.

In the mouse stool samples, the relative abundances of *Odoribacter*, *Mucispirillum*, *Escherichia*, *Bacteroides*, *Butyricicoccus*, *Butyricicoccus pullicaecorum*, *Mucispirillum schaedleri*, *Helicobacter hepaticus,* and *Bacteroides acidifaciens* were higher in the *db/db + PBS* mice than in the *db/m + PBS* mice. These bacteria levels were downregulated in the *db/db + KNGT* mice after six weeks of FMT. In contrast, the relative abundances of *Sutterella*, *Dorea*, *Coprococcus*, *Clostridium*, *Bacteroides uniformis,* and *Ruminococcus gnavus* were lower in the *db/db + PBS* mice. Interestingly, the levels of these bacteria were upregulated in the *db/db + KNGT* mice after six weeks of FMT (Figure 3(B,C), and Tables 6 and 7).

### Verification of target bacteria based on 16S rRNA gene sequencing

**Figure 4.**
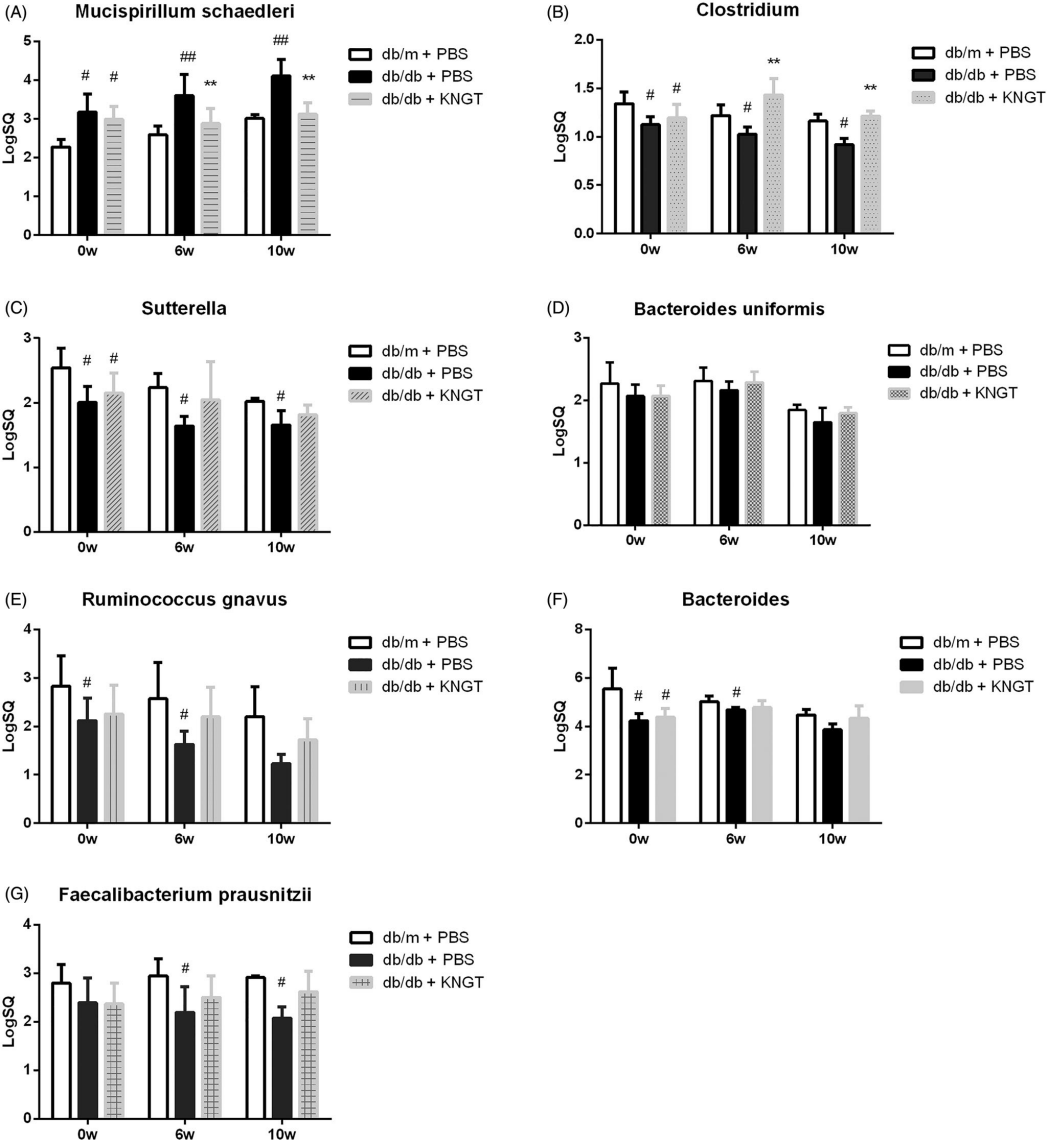
Quantification of gut bacteria in each group over time. SQ represents the starting template quantity of *Mucispirillum schaedleri* (A), *Clostridium* (B), *Sutterella* (C), *Bacteroides uniformis* (D), *Ruminococcus gnavus* (E), *Bacteroides* (F) and *Faecalibacterium prausnitzii* (G). On the horizontal axis, 0w, 6w, and 10w represent 0, 6 and 10 weeks after FMT-KNGT intervention. Data were analyzed using one-way ANOVA. #*p* < 0.05 and ##*p* < 0.01 compared to db/m + PBS mice. **p < 0.01 compared to db/db + PBS mice.

Based on 16S rRNA gene sequencing data (Figure 3(B,C)), we verified the target bacteria by RT-qPCR (Figure 4, Table 8). The RT-qPCR analysis showed that the *Mucispirillum schaedleri* levels in the faeces of *db/db + PBS* mice were significantly higher compared to the levels in the *db/m + PBS* mice (*p* < 0.01) (Figure 4(A)), while the *Clostridium* levels in the faeces of *db/db + PBS* mice were significantly lower than in the *db/m + PBS* mice (*p* < 0.05) (Figure 4(B)). *Sutterella*, *Bacteroides uniformis*, *Ruminococcus gnavus*, *Bacteroides*, and *Faecalibacterium prausnitzii* were reduced over time but not significantly (Figure 4(C–G)). When compared to the *db/db + PBS* mice, *Mucispirillum schaedleri* levels were significantly lower in *db/db + KNGT* mice over the FMT period (*p* < 0.01) (Figure 4(A)). In contrast, *Clostridium* levels were significantly higher over this time period (*p* < 0.01) (Figure 4(B)). There was also an upward trend in the *Bacteroides, Sutterella, Bacteroides uniformis*, *Ruminococcus gnavus*, and *Faecalibacterium prausnitzii* levels, but these changes did not reach statistical significance (Figure 4(C–G)).

**Table 8.** Effect of FMT- KNGT on the levels of target bacteria in the faeces of mice by RT-qPCR ($\bar{x}$ ± s, LogSQ).

Bacteria	Group	Time (Week)		
		0w	6w	10w
*Mucispirillum schaedleri*	db/m + PBS	2.28 ± 0.20	2.59 ± 0.22	3.01 ± 0.10
	db/db + PBS	3.17 ± 0.48#	3.60 ± 0.54##	4.11 ± 0.43##
	db/db + KNGT	2.98 ± 0.33#	2.88 ± 0.39**	3.12 ± 0.30**
*Clostridium*	db/m + PBS	1.34 ± 0.12	1.22 ± 0.11	1.16 ± 0.07
	db/db + PBS	1.12 ± 0.08#	1.02 ± 0.07#	0.92 ± 0.07#
	db/db + KNGT	1.19 ± 0.14#	1.43 ± 0.17**	1.21 ± 0.05**
*Sutterella*	db/m + PBS	2.54 ± 0.30	2.24 ± 0.22	2.02 ± 0.05
	db/db + PBS	2.00 ± 0.25#	1.64 ± 0.15#	1.66 ± 0.22#
	db/db + KNGT	2.15 ± 0.31#	2.05 ± 0.59	1.82 ± 0.15
*Bacteroides uniformis*	db/m + PBS	2.27 ± 0.34	2.31 ± 0.22	1.84 ± 0.09
	db/db + PBS	2.07 ± 0.18	2.16 ± 0.14	1.65 ± 0.23
	db/db + KNGT	2.07 ± 0.17	2.29 ± 0.17	1.79 ± 0.09
*Ruminococcus gnavus*	db/m + PBS	2.83 ± 0.63	2.57 ± 0.75	2.20 ± 0.61
	db/db + PBS	2.12 ± 0.46#	1.62 ± 0.28#	1.23 ± 0.20
	db/db + KNGT	2.25 ± 0.60	2.20 ± 0.61	1.72 ± 0.43
*Bacteroides*	db/m + PBS	5.54 ± 0.86	5.02 ± 0.23	4.47 ± 0.23
	db/db + PBS	4.23 ± 0.31#	4.68 ± 0.10#	3.86 ± 0.25
	db/db + KNGT	4.37 ± 0.36#	4.78 ± 0.29	4.34 ± 0.51
*Faecalibacterium prausnitzii*	db/m + PBS	2.80 ± 0.38	2.94 ± 0.36	2.91 ± 0.04
	db/db + PBS	2.39 ± 0.51	2.19 ± 0.53#	2.08 ± 0.23#
	db/db + KNGT	2.37 ± 0.43	2.50 ± 0.44	2.62 ± 0.43

SQ represents the starting template quantity. Data were analyzed using one-way ANOVA. #*p* < 0.05; ##*p <* 0.01 compared to *db/m + PBS* mice; **p < 0.01 compared to *db/db + PBS* mice. 0w: *n* = 6, 6w: *n* = 6, 10w: *n* = 3.

### Changes in faecal SCFA levels upon FMT intervention

**Figure 5.**
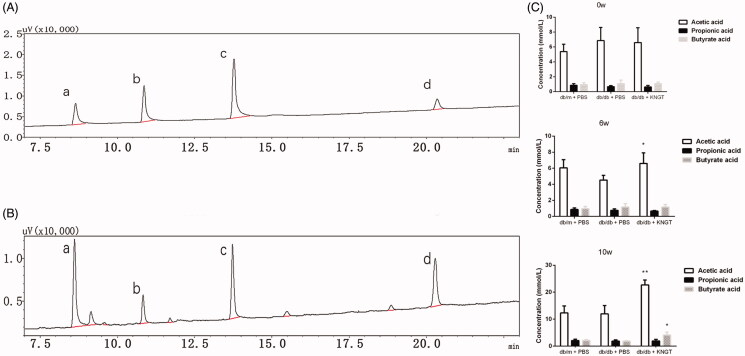
Gas chromatography comparisons between SCFA standards (A) and faecal samples (B) a = acetic acid, b = propionic acid, c = butyric acid, d = 2-ethyl butyric acid. SCFA data at 0, 6 and 10 weeks for faecal samples from mice after FMT-KNGT treatment (C). Data were analyzed using one-way ANOVA. *p < 0.05, **p < 0.01 compared to db/db + PBS mice.

Acetate, propionate and butyrate levels in faecal samples were quantified by GC (Figure 5(A,B)). Over time, faecal acetic acid levels were significantly increased in *db/db + KNGT* mice when compared to *db/db + PBS* mice (*p* < 0.05) (Figure 5(C), Table 9). Butyric acid levels were significantly increased after ten weeks of FMT in *db/db + KNGT* mice compared to *db/db + PBS* mice (*p* < 0.05) (Figure 5(C), Table 9). However, we observed no differences in the propionate levels across the three groups at any of the time points (Figure 5(C), Table 9).

**Table 9.** Concentrations of acetic acid, propionic acid and butyrate acid in faecal samples of mice ($\bar{x}$  ± s, mmol/L).

SCFAs	Group	Time (Week)		
		0w	6w	10w
Acetic acid	db/m + PBS	5.39 ± 0.98	6.05 ± 1.03	12.32 ± 2.60
	db/db + PBS	6.86 ± 1.77	4.52 ± 0.59	11.97 ± 3.06
	db/db + KNGT	6.60 ± 1.98	6.60 ± 1.32*	22.68 ± 1.82**
Propionic acid	db/m + PBS	0.85 ± 0.22	0.87 ± 0.19	2.09 ± 0.47
	db/db + PBS	0.67 ± 0.15	0.75 ± 0.18	1.87 ± 0.47
	db/db + KNGT	0.64 ± 0.21	0.66 ± 0.07	1.93 ± 0.60
Butyric acid	db/m + PBS	0.95 ± 0.25	0.97 ± 0.27	2.13 ± 0.27
	db/db + PBS	1.07 ± 0.48	1.17 ± 0.41	1.87 ± 0.15
	db/db + KNGT	1.09 ± 0.20	1.17 ± 0.30	4.13 ± 1.09*

Data were analyzed using one-way ANOVA. *p < 0.05; **p < 0.01 compared to *db/db + PBS* mice. 0w: *n* = 6, 6w: *n* = 6, 10w: *n* = 3.

### Expression of GPR43 and GLP-1 mRNA during FMT treatment

**Figure 6.**
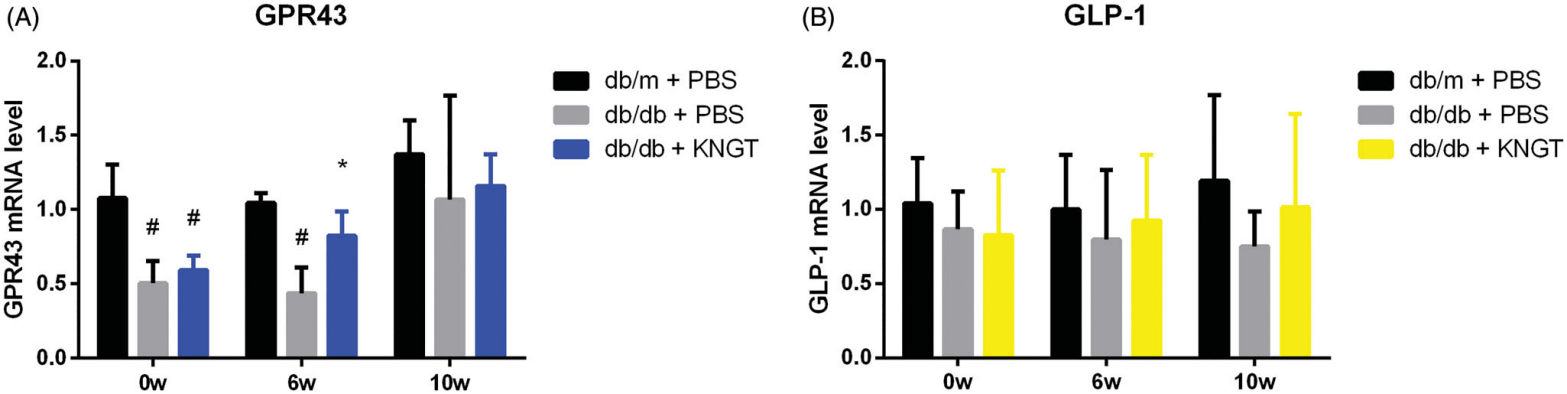
GPR43 mRNA (A) and GLP-1 (B) levels in colon tissues from each mouse group after FMT-KNGT at 0, 6 and 10 weeks. Data were analyzed using one-way ANOVA. #*p* < 0.05 compared to db/m + PBS mice. *p < 0.05 compared to db/db + PBS mice.

GPR43 and GLP-1 mRNA levels were lower in the colons from *db/db + PBS* mice than *db/m + PBS* mice. FMT-KNGT for six weeks significantly increased GPR43 expression levels in the colons of *db/db + KNGT* mice (*p* < 0.05) (Figure 6(A), Table 10). However, no statistical differences were observed after ten weeks of FMT. FMT-KNGT increased GLP-1 expression levels over time in the colons of the *db/db + KNGT* mice (Figure 6(B), Table 10).

**Table 10.** GPR43 and GLP-1 mRNA expression in colonic tissue of mice ($\bar{x}$ ± s, *n* = 3).

mRNA	Group	Time (week)		
		0w	6w	10w
GPR43	db/m + PBS	1.08 ± 0.23	1.05 ± 0.07	1.37 ± 0.23
	db/db + PBS	0.50 ± 0.15#	0.44 ± 0.17#	1.07 ± 0.70
	db/db + KNGT	0.60 ± 0.10#	0.82 ± 0.16*	1.16 ± 0.21
GLP-1	db/m + PBS	1.04 ± 0.30	1.00 ± 0.37	1.19 ± 0.58
	db/db + PBS	0.87 ± 0.25	0.80 ± 0.47	0.75 ± 0.24
	db/db + KNGT	0.83 ± 0.43	0.92 ± 0.44	1.01 ± 0.63

Data were analyzed using one-way ANOVA. #*p* < 0.05 compared to *db/m + PBS* mice; *p < 0.05 compared to *db/db + PBS* mice.

### GLP-1 protein expression during FMT treatment

**Figure 7.**
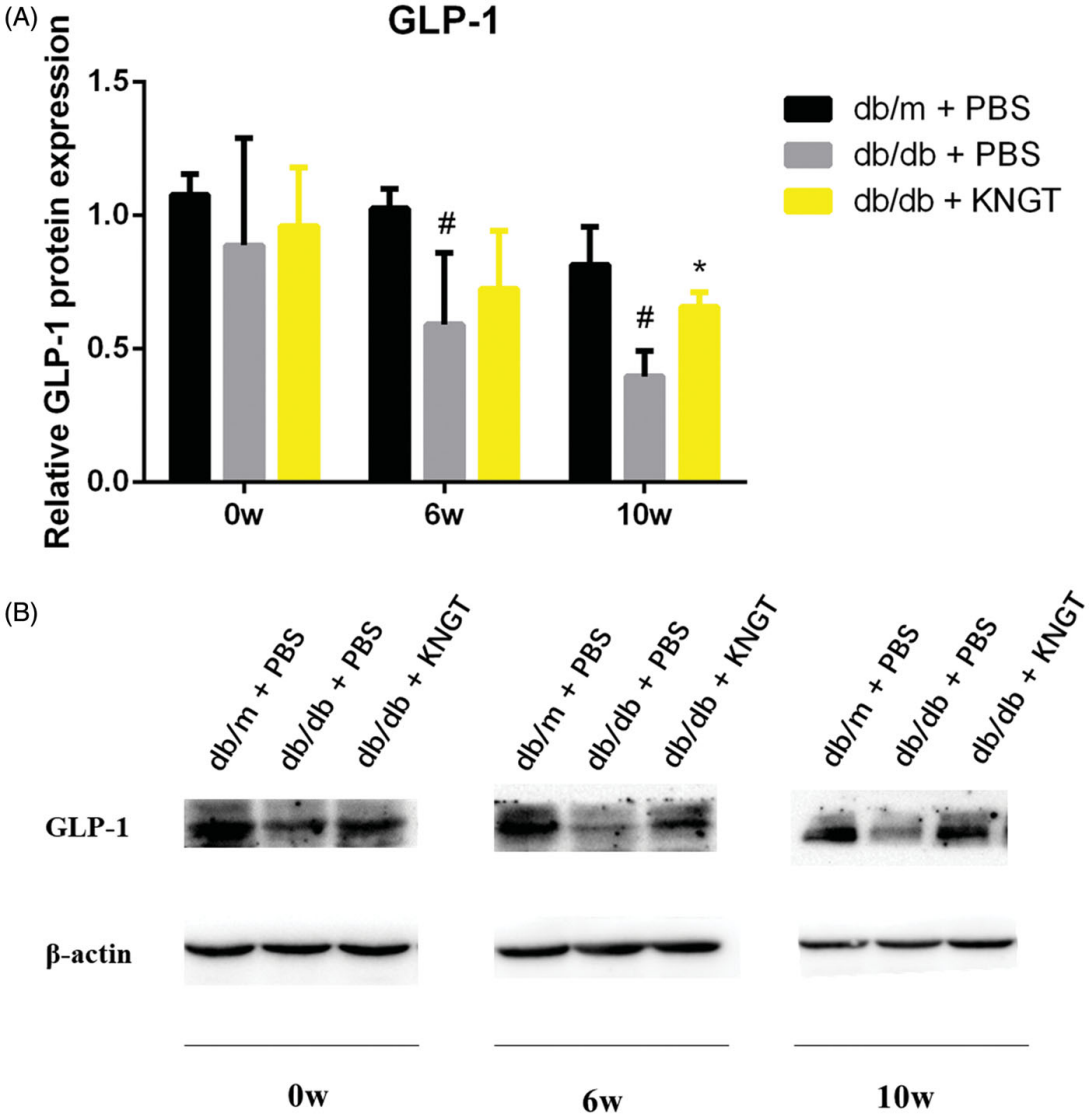
GLP-1 protein expression in the colon tissue of each mouse group after FMT-KNGT at 0, 6 and 10 weeks. The grey values were analyzed by ImageJ. Data are presented as the mean ± SD values. *p < 0.05 compared to db/m + PBS mice. #*p* < 0.05 compared to db/db + PBS mice.

GLP-1 protein expression was significantly lower in the colon of the *db/db + PBS* group over time when compared to the *db/m + PBS* group (*p* < 0.05). FMT-KNGT over a 10-week period significantly increased GLP-1 expression in the colon of *db/db + KNGT* mice (*p* < 0.05**)** (Figure 7(A), Table 11).

**Table 11.** Protein expression of GLP-1 in colonic tissue of mice ($\bar{x}$ ± s, *n* = 3).

Protein expression	Group	Time (Week)		
		0w	6w	10w
GLP-1	db/m + PBS	1.08 ± 0.08	1.03 ± 0.07	0.82 ± 0.14
	db/db + PBS	0.89 ± 0.40	0.59 ± 0.27#	0.40 ± 0.09#
	db/db + KNGT	0.96 ± 0.22	0.73 ± 0.22	0.62 ± 0.20*

Data were analyzed using one-way ANOVA. *p < 0.05 compared to *db/m + PBS* mice; #*p* < 0.05 compared to *db/db + PBS* mice.

### Correlation analysis

**Figure 8.**
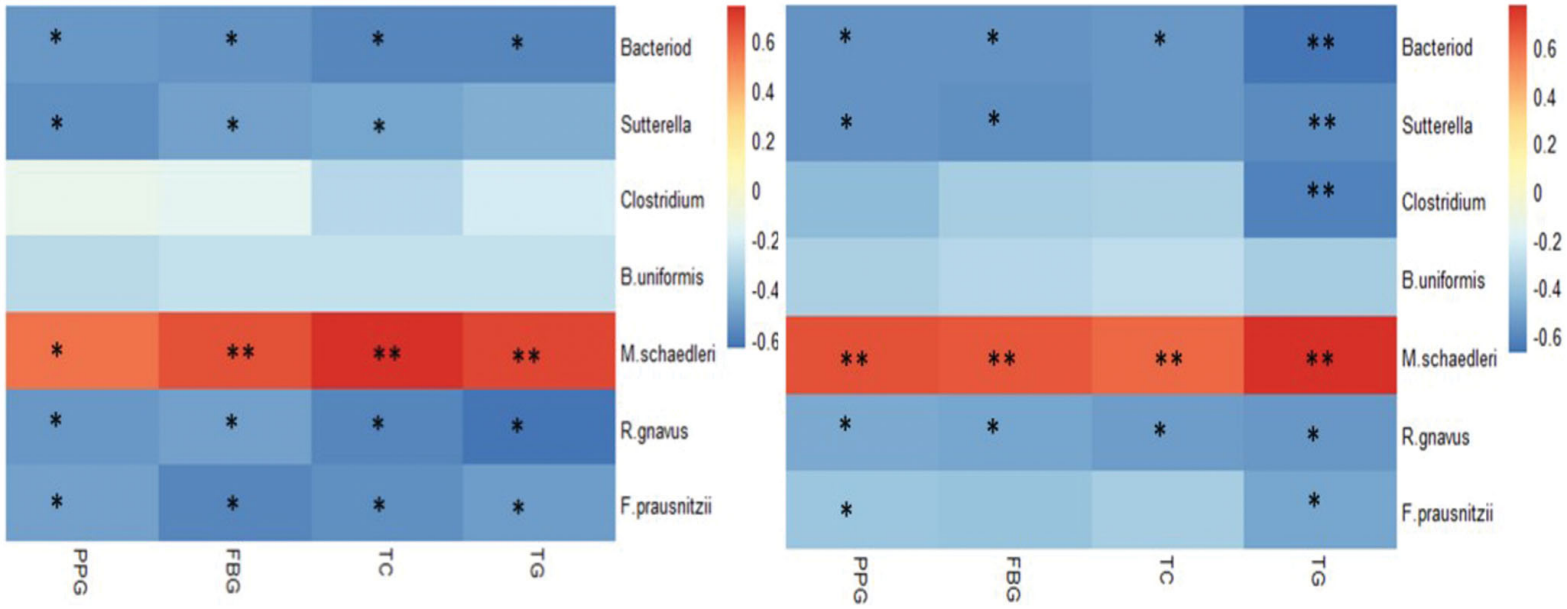
Correlations between faecal target bacteria and plasma glycolipid levels in three groups mice treated with FMT-KNGT for 6 weeks (A) and 10 weeks (B). R value is between 0.6∼-0.6; *p < 0.05, **p < 0.01.

Pearson correlation analysis for the seven target bacteria and glycolipid metabolism parameters after 6 weeks of FMT-KNGT showed that *Bacteroides*, *Faecalibacterium prausnitziiand,* and *Ruminococcus gnavus* levels were negatively correlated with FBG, PPG, TC and TG levels. *Sutterella* levels were also negatively correlated with PPG, FBG, and TC levels. In contrast, *Mucispirillum schaedleri* levels were positively correlated with FBG, PPG, TC, and TG levels (Figure 8(A)).

Pearson correlation analysis after ten weeks of FMT-KNGT showed that the expression levels of *Bacteroides* and *Ruminococcus gnavus* were still negatively correlated with FBG, PPG, TC, and TG levels, and *Sutterella* levels were negatively correlated with PPG, FBG, and TG levels. *Clostridium* levels were negatively correlated with TG, and *Faecalibacterium prausnitzii* levels were negatively correlated with PPG and TG levels. Moreover, *Mucispirillum schaedleri* levels were positively correlated with FBG, PPG, TC, and TG levels (Figure 8(B)).

## Discussion and conclusions

More and more evidence suggests that intestinal microbiota is a causative factor in T2DM (Tong et al. 2018). Therefore, the intestinal microbiota is a promising target for disease control. We determined the species composition of KNGT donor faecal samples and mouse faecal samples after FMT-KNGT by 16S rRNA gene sequencing. Twenty genera were identified. *Prevotella* accounted for 78.04% of the bacteria at the genus level (Table 5). It was previously reported (Kovatcheva-Datchary et al. 2015) that the oral administration of *Prevotella or Prevotella copri* bacteria from normal faeces to C57 BL/6 mice for seven days improved glucose metabolism levels. However, we did not observe such a phenomenon in our study. After six weeks of FMT-KNGT in *db/db* mice, the relative abundance of *Prevotella* in the three groups was not statistically different.

Interestingly, nine genera of *Odoribacter*, *Mucispirillum*, *Escherichia*, *Bacteroides*, *Butyricicoccus*, *Sutterella*, *Dorea*, *Coprococcus,* and *Clostridium* and six strains of *Butyricicoccus pullicaecorum*, *Mucispirillum schaedleri*, *Helicobacter hepaticus*, *Bacteroides acidifaciens*, *Bacteroides uniformis,* and *Ruminococcus gnavus* were changed among the three groups (Tables 6 and 7). Rawls et al. (2006) showed that after transplanting mouse and zebrafish microbiota into germ-free zebrafish and mice, the hosts supported a complex foreign microbial consortium by shaping its composition. Similarly, our results demonstrated that after foreign microbiota were transplanted into *db/db* mice, the community composition in the mice changed. The changes were not towards the composition of the donor community; however, under the actions of the recipient host, some bacteria with low relative abundance changed (e.g., *Bacteroides uniformis*). Seven bacteria were evaluated by RT-qPCR. We found that *Bacteroides*, *Sutterella*, *Clostridium*, *Bacteroides uniformis*, and *Ruminococcus gnavus* levels decreased after FMT-KNGT over time, while the *Mucispirillum schaedleri* content increased with time. *Bacteroides*, *Clostridium,* and *Ruminococcus gnavus* are acetic acid-producing bacteria, and *Bacteroides uniformis* and *Faecalibacterium prausnitzii* are butyric acid-producing (Koh et al. 2016). *Mucispirillum schaedleri* can induce intestinal inflammation in doubly-deficient Nod2/Cybb mice (Caruso et al. 2019). Moreover, *Clostridium* is depleted in individuals with pre-diabetes, and these levels are negatively correlated with fasting levels of glucose and triacylglycerol, insulin resistance estimates, inflammation and adiposity (Allin et al. 2018). Butyric acid is a metabolite of *Bacteroides uniformis* that improves the metabolic and immune dysfunction caused by gut microbiota imbalance in obese mice (Gauffin Cano et al. 2012). Furthermore, intestinal SCFAs are associated with T2DM, and glycolipid levels can be regulated by SCFAs (Li et al. 2017).

Therefore, we observed that after six weeks of FMT-KNGT treatment, acetic acid levels increased significantly in the *db/db + KNGT* group, whereas butyric acid levels significantly increased after ten weeks of treatment, suggesting that bacterial metabolites could significantly change in response to changes in the microbiota in relation to the intervention time. A previous study showed that SCFAs were GPR43 agonists to stimulate GLP-1 and PYY secretion from L cells, which could improve dysfunctional glucose and lipid metabolism and insulin resistance (Koh et al. 2016; Wang et al. 2020). We found GPR43 mRNA expression and GLP-1 protein expression levels in the colons of *db/db* mice increased after six weeks of FMT-KNGT treatment and were related to the SCFA data. These results suggested that promoting GLP-1 secretion might be dependent on the upregulation of GPR43 by SCFAs. Thus, our data demonstrated that FMT from Kazaks could promote GLP-1 secretion induced by the SCFAs by regulating gut microbiota in *db/db* mice. However, one limitation of our study was the use of only FMT-KNGT. The effects of human faecal transplantation from Uygur and Han nationalities with normal glucose tolerance on T2DM should be included. This deficiency will be addressed in future studies to investigate the FMT-KNGT mechanisms for improving dysfunctional glucose and lipid metabolism.

One of the possible mechanisms for the ability of FMT-KNGT to improve dysfunctional glucose and lipid metabolism is that by increasing the diversity of the gut microbiota and affecting the different microbial species in the intestines of mice, the level of bacteria producing SCFAs could increase, resulting in an increased SCFA content in the faeces of mice. This increase might activate the GPR43/GLP-1 pathway and increase GLP-1 expression in the colon, thus, regulating dysfunctional glucose and lipid metabolism.

## Supplementary Material

Supplemental Material

Supplemental Material

Supplemental Material

Supplemental Material

Supplementary_5.docx

Supplementary_4.docx
