# Supplementary material for: Kazak faecal microbiota transplantation induces short-chain fatty acids that promote glucagon-like peptide-1 secretion by regulating gut microbiota in db/db mice
Source: Pharm Biol. 2021 Aug 15;59(1):1075–85. doi: 10.1080/13880209.2021.1954667 (PMC8366640; doi:10.1080/13880209.2021.1954667)
Supplement: Supplemental Material [file IPHB_A_1954667_SM0921.pdf]

The PCR product sequences of target bacteria.

Target bacteria	PCR product sequences
Clostridium	GGGGGGGAGCCCTTCAGGGCGGAAGACATGTGGTGCATGGTTGT CGTCAGCTCGTGTCGTGAGATGTTGGGTTAAGTCCCGCACAG
Bacteroides	GTGGGTAGACGTCGGGTTGTAACTGCTTTTATATCGGAATTAAAA GGGGCACCTGGGCCTTTTTGCATGTACCTTATGAATAAGGATCGG CTAACTCCGTGCCAGCAGCCGCGGTAATACGGAGGATCCGAGCG TTATCCGGATTTATTGGGTTTAAAGGGAGCGTAGATGGGTTGTTA AGTCAGTTGTGAAAGTTTGCGGA
Sutterella	AAGGGGGTAGATGACGTCATCCCACCTTCCTCCGGTTTGTCACCG GCAGTCTCACTAGAGTGCCCTTTCGTAGCAACTAGTGACAAGGGT TGCGCTCGTTGCGGGACTTAACCCAACATCTCACGACACGAGCTG ACGACAGCCATGCAGCACCTGTGTTCAGACGCCCTTGCGGGCACA CTCTCATTACAAAAGCTTCTCTGACATGTCAAGGCTAGGTAAGGT TTTTCGCGA
Mucispirillum schaedleri	AGCCTTGGGGCAGTAGGCGGTGTTGTAGTCATTAGTCAAAGACTA GAGCTCAACTTTAGTAAGGCTAGTGATACTATAATACTAGAGTAT CAGAGAGGATTGCAGAATTCCTGGTGTAGCGGTGAAATGCGTAG ATATCAGGAGGAATACCGTTAGCGAAGGCGGCAATCTGGCTGGA
Ruminococcus gnavus	GCGGCATTGCCTGGTGTTCTCCTATATCTACGCATTTCACCGCTAC ACTAGGAATTCCACTTACCTCTCCGACACTCTAGCCTGACAGTTC CAAATGCAGTCC
Bacteroides uniformis	GGGGGGCATCTTGACCTATCCATCGATGCCTTGGTGGGCCGTTAC CCCGCCAACAAGCTAATGGAACGCATCCCCATCGATGACCGAAA TTCTTTAATAGTTCTACCATGCG
Faecalibacterium prausnitzii	GGACAAGGTGGTCGGCCACAAGGCGACGATCGGTAGCCAGGACT GAGAGGTTGAACGGCCACATTGGGACTGAGACACGGCCCAGACT CCTACGGGAGGCAGCAGTGGGGAATATTGCACAATGGGGGAAAC CCTGATGCAGCAACGCCGCGTGGAGGAAGTAGGTCTTCAGAAAA T
