# Supplementary material for: Kazak faecal microbiota transplantation induces short-chain fatty acids that promote glucagon-like peptide-1 secretion by regulating gut microbiota in db/db mice
Source: Pharm Biol. 2021 Aug 15;59(1):1075–85. doi: 10.1080/13880209.2021.1954667 (PMC8366640; doi:10.1080/13880209.2021.1954667)
Supplement: Supplemental Material [file IPHB_A_1954667_SM0919.pdf]

Informed Consent Form

Dear donor:

We are inviting you to participate in a study on the molecular mechanism of intestinal microbiome causing type 2 diabetes mellitus(T2DM). This study requires you to participate in our professional questionnaire and provide stool samples. The personal information provided from you is only used for scientific research purposes and will be handled in accordance with national regulations. We guarantee that all your personal data and observation records will be kept confidential only for scientific research.

Purpose of research

To find the species and structure ratio of gut microbiota that are strong correlation to T2DM, the fecal commensal bacteria solution provided by the Chinese Kazak individuals will transplant into insulin resistant mice in intestinal microbiome of Chinese Kazak individuals and T2DM research. Furthermore, the target microbes are further cultured and metabolites are analyzed. It laid a foundation for the study of gut microbiota of Chinese kazak individuals and mechanism of T2DM.

We sincerely hope that you can participate in this research. It is a project of the national natural science foundation of China. The study has been approved by the ethics committee of the first clinical medical school of Xinjiang Medical University. Meanwhile it complies with the principles of the declaration of Helsinki and medical ethics. This study is based entirely on the principle of voluntary participation.

Contents of research

From the detected gut microbiota in the fecal samples of Chinese kazak donors, to analyze the correlation between the species and structure of gut microbiota and intermediate phenotype of T2DM.

Risks

When you participate in this study, we need to collect your fecal samples for testing. When taking samples, we guarantee safety and efficiency. In addition to your rights and interests in the physical examination process, we ensure the standardization and routinization of the sampling process. Your health is always our priority.

When you participate in this study, a comprehensive physical examination is required. These will take up some of your time, and may also cause you trouble or inconvenience; Some sample donors may feel uncomfortable during the examination, which can be improved after treatment by doctors.

If you experience any discomfort during the examination, regardless of whether it is related to the study, you should inform your examining physician promptly, he/she will make a judgment and give appropriate medical treatment.

Benefits

For all the sample donors who participate in the study, our research group will provide you free physical examination with designated items by us in the 3A hospital of this city, and offer you feedback in time.

Confidentiality

• Respect the privacy of the donor. Without donor's permission, do not disclose information of the sample donor's examination and the results of the examination to any other person at any time, other than the authorized investigator, the examination results and data collected are for scientific research only.

• You are also not free to disclose the contents of our research to any third party.

I, the undersigned, confirm that (please tick box as appropriate):

1	I have read and understood the information about the project, as provided in the Information Sheet dated Dec. 11th. 2017	☑
2	I have been given the opportunity to ask questions about the project and my participation.	☑
3	I voluntarily agree to participate in the project.	☑
4	I understand I can withdraw at any time without giving reasons and that I will not be penalised for withdrawing nor will I be questioned on why I have withdrawn.	☑
5	The procedures regarding confidentiality have been clearly explained (e.g. use of names, pseudonyms, anonymization of data, etc.) to me.	☑
6	If applicable, separate terms of consent for interviews, audio, video or other forms of data collection have been explained and provided to me.	☑
7	The use of the data in research, publications, sharing and archiving has been explained to me.	☑
8	I understand that other researchers will have access to this data only if they agree to preserve the confidentiality of the data and if they agree to the terms I have specified in this form.	☑
9	Select only one of the following:<br><br>I would like my name used and understand what I have said or written as part of this study will be used in reports, publications and other research outputs so that anything I have contributed to this project can be recognised.	☑
	I do not want my name used in this project.	☐
10	I, along with the Researcher, agree to sign and date this informed consent form.	☑

Participant:

拜克扎提·昂沙行

Bekzat • Ongsahong — 拜克扎提昂沙行 Bekzat Ongsahong — Dec. 11th. 2017

Name of Participant — Signature — Date

Researcher:

韩 雪 Xue Han — 韩雪 Xue Han — Dec. 12th. 2017

Name of Researcher — Signature — Date
