# Supplementary material for: Kazak faecal microbiota transplantation induces short-chain fatty acids that promote glucagon-like peptide-1 secretion by regulating gut microbiota in db/db mice
Source: Pharm Biol. 2021 Aug 15;59(1):1075–85. doi: 10.1080/13880209.2021.1954667 (PMC8366640; doi:10.1080/13880209.2021.1954667)
Supplement: Supplemental Material [file IPHB_A_1954667_SM0915.pdf]

Amend Letter of Ethics Committee of First Affiliated Hospital of Xinjiang Medical University

Approval No: 20140212-113 Approval data: February 17th, 2014

Project title: Interaction of genetic and environmental factors in type 2 diabetes mellitus [U1403322]

Project leader: Dr. Linlin Li

Approval comments:

The Ethics Committee of the hospital considered the project to meet the ethical requirements through review and approved the application.

Signature of the Chair of the Ethics Committee: Jian Liu

Date: February 17th, 2014
