# Supplementary figures and images for: Kazak faecal microbiota transplantation induces short-chain fatty acids that promote glucagon-like peptide-1 secretion by regulating gut microbiota in db/db mice
Source: Pharm Biol. 2021 Aug 15;59(1):1075–85. doi: 10.1080/13880209.2021.1954667 (PMC8366640; doi:10.1080/13880209.2021.1954667)

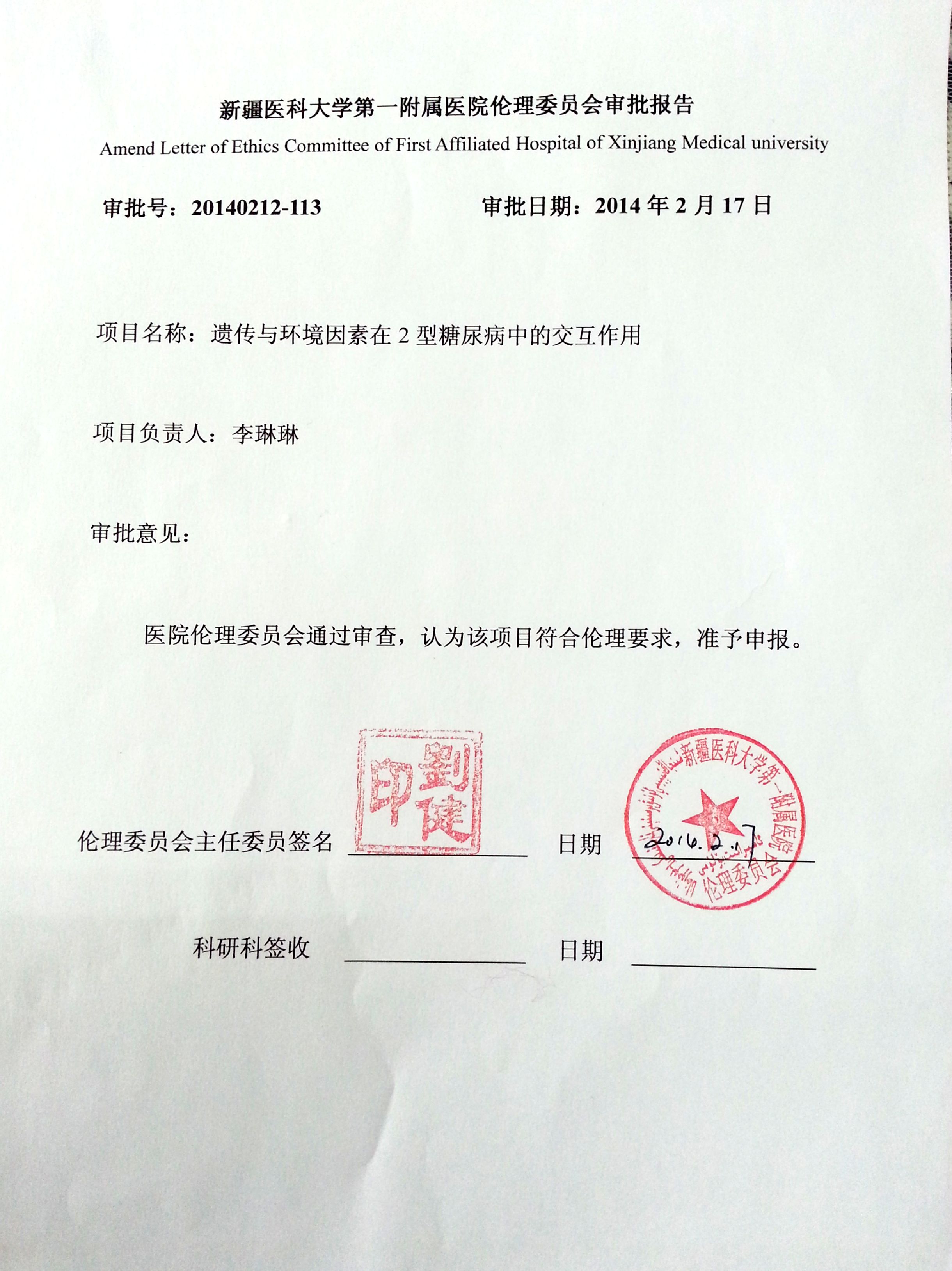

Supplement: Supplemental Material [file IPHB_A_1954667_SM0913.jpg]
