# Supplementary material for: Kazak faecal microbiota transplantation induces short-chain fatty acids that promote glucagon-like peptide-1 secretion by regulating gut microbiota in db/db mice
Source: Pharm Biol. 2021 Aug 15;59(1):1075–85. doi: 10.1080/13880209.2021.1954667 (PMC8366640; doi:10.1080/13880209.2021.1954667)
Supplement: Supplementary_5.docx [file IPHB_A_1954667_SM1924.docx]

***Determination of maximal tolerable dose (MTD)***

Acute toxicity was observed in ICR mice [SCXK (Xinjiang) 2018-0002] fed with fecal microorganisms from KNGT donors (same as original manuscript). A total of 40 ICR mice with qualified SPF grade were selected, with a body weight of 18.2 ~ 22.0 g, equal to half male and half female. Each mouse was raised in the cage with 5 mice. Males and females were randomly divided into two groups according to sex and body weight: control group (normal saline phosphate buffer, PBS) and fecal suspension group (same as original manuscript), with 20 mice in each group. Before the experiment, ICR mice were fasted for 16 hours and given the maximum oral dose of PBS and fecal suspension at a dose of 40mL/kg. The fecal suspension was given once the day. Toxicity manifestations and characteristics, occurrence of toxic reactions, recovery time, and death time of mice in each group were observed and recorded within 0 ~ 4 h, observed once a day for 14 days.

The effects on general activities, animal poisoning symptoms and death: the control group and the fecal suspension group had normal behavior within 0 ~ 4 h after administration and no toxic reactions were observed. No abnormalities or animal deaths were observed for 14 consecutive days.
