# Supplementary material for: Kazak faecal microbiota transplantation induces short-chain fatty acids that promote glucagon-like peptide-1 secretion by regulating gut microbiota in db/db mice
Source: Pharm Biol. 2021 Aug 15;59(1):1075–85. doi: 10.1080/13880209.2021.1954667 (PMC8366640; doi:10.1080/13880209.2021.1954667)
Supplement: Supplementary_4.docx [file IPHB_A_1954667_SM1919.docx]

**Supplementary 4.**

**The PCR product sequences of target bacteria.**

| Target bacteria | PCR product sequences |
| --- | --- |
| *Clostridium* | GGGGGGGAGCCCTTCAGGGCGGAAGACATGTGGTGCATGGTTGTCGTCAGCTCGTGTCGTGAGATGTTGGGTTAAGTCCCGCACAG |
| *Bacteroides* | GTGGGTAGACGTCGGGTTGTAACTGCTTTTATATCGGAATTAAAAGGGGCACCTGGGCCTTTTTGCATGTACCTTATGAATAAGGATCGGCTAACTCCGTGCCAGCAGCCGCGGTAATACGGAGGATCCGAGCGTTATCCGGATTTATTGGGTTTAAAGGGAGCGTAGATGGGTTGTTAAGTCAGTTGTGAAAGTTTGCGGA |
| *Sutterella* | AAGGGGGTAGATGACGTCATCCCACCTTCCTCCGGTTTGTCACCGGCAGTCTCACTAGAGTGCCCTTTCGTAGCAACTAGTGACAAGGGTTGCGCTCGTTGCGGGACTTAACCCAACATCTCACGACACGAGCTGACGACAGCCATGCAGCACCTGTGTTCAGACGCCCTTGCGGGCACACTCTCATTACAAAAGCTTCTCTGACATGTCAAGGCTAGGTAAGGTTTTTCGCGA |
| *Mucispirillum schaedleri* | AGCCTTGGGGCAGTAGGCGGTGTTGTAGTCATTAGTCAAAGACTAGAGCTCAACTTTAGTAAGGCTAGTGATACTATAATACTAGAGTATCAGAGAGGATTGCAGAATTCCTGGTGTAGCGGTGAAATGCGTAGATATCAGGAGGAATACCGTTAGCGAAGGCGGCAATCTGGCTGGA |
| *Ruminococcus*  *gnavus* | GCGGCATTGCCTGGTGTTCTCCTATATCTACGCATTTCACCGCTACACTAGGAATTCCACTTACCTCTCCGACACTCTAGCCTGACAGTTCCAAATGCAGTCC |
| *Bacteroides*  *uniformis* | GGGGGGCATCTTGACCTATCCATCGATGCCTTGGTGGGCCGTTACCCCGCCAACAAGCTAATGGAACGCATCCCCATCGATGACCGAAATTCTTTAATAGTTCTACCATGCG |
| *Faecalibacteriumprausnitzii* | GGACAAGGTGGTCGGCCACAAGGCGACGATCGGTAGCCAGGACTGAGAGGTTGAACGGCCACATTGGGACTGAGACACGGCCCAGACTCCTACGGGAGGCAGCAGTGGGGAATATTGCACAATGGGGGAAACCCTGATGCAGCAACGCCGCGTGGAGGAAGTAGGTCTTCAGAAAAT |
